# Surface reconstruction of cobalt phosphide nanosheets by electrochemical activation for enhanced hydrogen evolution in alkaline solution†
†Electronic supplementary information (ESI) available: Experimental details, XRD, SEM, TEM, XPS, and electrochemical measurements. See DOI: 10.1039/c8sc04589e


**DOI:** 10.1039/c8sc04589e

**Published:** 2018-12-06

**Authors:** Liang Su, Xiangzhi Cui, Ting He, Liming Zeng, Han Tian, Yiling Song, Kai Qi, Bao Yu Xia

**Affiliations:** a Key Laboratory of Material Chemistry for Energy Conversion and Storage (Ministry of Education) , Hubei Key Laboratory of Material Chemistry and Service Failure , Wuhan National Laboratory for Optoelectronics , School of Chemistry and Chemical Engineering , Huazhong University of Science and Technology (HUST) , 1037 Luoyu Road , Wuhan 430074 , PR China . Email: byxia@hust.edu.cn; b State Key Laboratory of High Performance Ceramics and Superfine Microstructures , Shanghai Institute of Ceramics , Chinese Academy of Sciences , Shanghai 200050 , PR China . Email: cuixz@mail.sic.ac.cn; c University of the Chinese Academy of Sciences , Beijing 100049 , PR China

## Abstract

Transition metal phosphides exhibit promising catalytic performance for the hydrogen evolution reaction (HER); however their surface structure evolution during electrochemical operation has rarely been studied. In this work, we investigate the surface reconstruction of CoP nanosheets by an *in situ* electrochemical activation method. After remodeling, CoP nanosheets experience an irreversible and significant evolution of the morphology and composition, and low-valence Co complexes consisting of Co(OH)_*x*_ species are formed on the surface of CoP nanosheets, and they largely accelerate the dissociation of water. Benefiting from the synergistic effect of CoP and Co(OH)_*x*_, the working electrode shows a remarkably enhanced HER activity of 100 mV at 10 mA cm^–2^ with a Tafel slope of 76 mV dec^–1^, which is better than that of most transition metal phosphide catalysts. This work would provide a deep understanding of surface reconstruction and a novel perspective for rational design of high performance transition metal phosphide electrocatalysts for water related electrolysis.

## Abstract 2

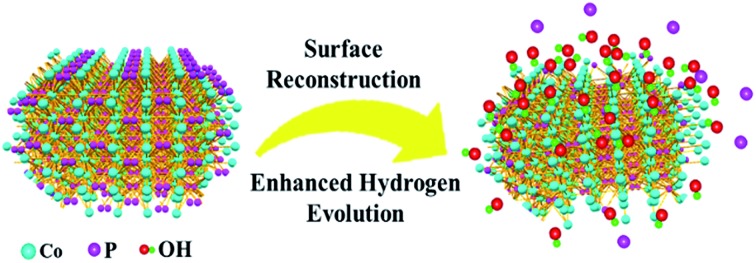
The surface reconstruction of cobalt phosphide nanosheets is investigated by an *in situ* electrochemical strategy for enhanced hydrogen evolution.

## 

Hydrogen is widely recognized as a first-class energy source compared to fossil fuels, owing to its excellent characteristics including outstanding gravimetric energy density, high energy conversion efficiency and renewability.^1^,^2^ Electrochemical water splitting is an ideal method to produce hydrogen with high purity.^3^ In order to accelerate the kinetics of the hydrogen evolution reaction (HER) and decrease the reaction overpotential, numerous efforts have been made to explore efficient electrocatalysts.^4^,^5^ Although Pt-based materials are presently the most active for the HER with high current density at low overpotential, their utilization on a large scale is unrealistic due to the high cost and limited resources.^6^ To replace the high cost Pt-based materials, lots of earth-abundant transition metal compounds have been extensively investigated.^7^,^8^ Metal chalcogenides,^9^ selenides^10^ and phosphides^11^ have been demonstrated as promising candidates for HER electrocatalysts, as the introduction of non-metallic elements can effectively regulate the hydrogen adsorption and achieve a delicate balance of H_ads_ recombination and OH^–^ desorption.^12^,^13^ Nevertheless, their real active surface would suffer from elemental leaching and thus result in surface remodeling during practical operation.^14^–^16^ It is therefore necessary and significant to understand the real evolution and reaction mechanism; however, close attention has not been paid to this process.

The typical HER process involves the formation of hydrogen intermediates followed by molecular hydrogen generation.^17^ However, the capability of water dissociation is the prior requirement for water splitting following the subsequent adsorption of hydrogen and desorption of hydroxyl ions.^18^,^19^ Various classic strategies have been developed to increase the water dissociation by providing sufficient proton sources and capability,^17^ such as adjusting the electronic structure through metal doping^20^,^21^ or defect engineering,^9^,^22^ and even surface modification by amino-containing functional groups to enhance the concentration of surface protons.^23^ However, such design principles have mainly focused on the energy balance of the absorption/desorption of hydrogen according to the well-known volcano plot,^13^ while the desorption of hydroxyl ions and water dissociation, and the nature of the proton donor in alkaline electrolysis, have rarely been considered.^24^,^25^

Studies on the water–gas shift reaction have found metal (hydr)oxides to be effective for cleaving the H–OH bond due to the high capability of water dissociation; thus, constructing a hybrid surface of metal (hydr)oxides on metal phosphides may enhance the kinetics for hydrogen production.^26^–^29^ Herein, we develop an *in situ* electrochemical method to engineer the surface of cobalt phosphides and achieve a stable Co(OH)_*x*_@CoP hybrid as a robust and efficient electrocatalyst for the HER. The as-obtained Co(OH)_*x*_@CoP demonstrates a remarkably enhanced HER activity with an overpotential of 100 mV at 10 mA cm^–2^. We attribute the enhanced activity to the synergistic effect of the optimized surface/interface of the hybrid Co(OH)_*x*_@CoP species formed, which promotes water dissociation through the increased concentration of surface hydroxyl species. This work offers a new way to enhance the activity through surface engineering by effective electrochemical reconstruction, which would provide useful understanding of the surface evolution and reconstruction of catalysts and would offer a useful strategy for the design and preparation of highly efficient electrocatalysts for practical application.

CoP nanosheets are prepared by a topological transformation using Co-oxides as the precursor followed by a phosphidation process in a reductive environment (Fig. S1†). X-ray diffraction (XRD) patterns indicate the successful transformation of the cobalt oxide precursor to crystalline CoP (PDF 29-0497) (Fig. 1a). The scanning electron microscopy (SEM) image of CoP products demonstrates the interconnected nanosheet morphology (Fig. 1b). Fig. 1c clearly shows an average size of 200 nm width and less than 5 nm thickness for these nanosheets. Moreover, the elements including Co, P, and O are homogeneously dispersed according to the elemental mapping images (Fig. S2c†). From the corresponding energy-dispersive X-ray spectrometry (EDX) result (Fig. S2e†), the elemental content of Co and P is 21.1 at% and 19.2 at%, respectively, demonstrating that the stoichiometric ratio of Co to P is close to 1 : 1. Transmission electron microscopy (TEM) images present a nanosheet structure (Fig. 1d), while the thickness is estimated to be ∼4 nm, consistent with the SEM observation (Fig. 1c). The well-resolved lattice fringes with interplanar spacings of 0.24 nm and 0.21 nm correspond to the (210) and (201) planes of crystalline CoP, respectively (Fig. 1f).

**Fig. 1:**
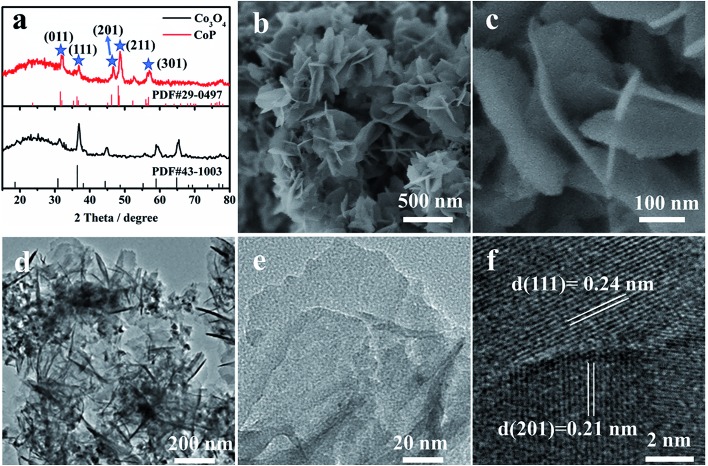
(a) XRD patterns, (b and c) SEM images, (d and e) TEM images and (f) HRTEM images of CoP nanosheets.

The surface reconstruction is realized through an *in situ* electrochemical chronopotentiometry method at a constant current density of 20 mA cm^–2^ for 10 hours until the potentials become stable. SEM images demonstrate that the CoP sample experienced an obvious change after the activation process, denoted as CoP-A. Generally, the CoP-A sample still retains the nanosheet morphology, while the surface becomes rougher compared to the smooth surface of the initial CoP nanosheets (Fig. 2a). Apparently, some small clusters are formed on the surface of the CoP-A sample. Fig. 2b shows slightly thick nanosheets with an average width of 200 nm and a thickness of ∼5 nm, which are similar to the CoP nanosheets. TEM images further show that CoP-A largely retains the morphology of two-dimensional nanosheets although some of them are assembled together in a certain direction due to the material's corrosion or reorganization (Fig. 2d). Interestingly, according to the high resolution (HR)TEM in Fig. 2e, an interplanar spacing of ∼0.25 nm in the interior of nanosheets (region I) corresponds to the (211) plane of CoP (PDF#29-0497),^23^ while the interplanar spacing at the outer surface of nanosheets (region II) is narrower and distinctly different from that in the inner CoP lattice, suggesting the formation of new species on the CoP-A nanosheet surface. The element mapping images still show the uniform distribution of composition in CoP-A nanosheets (Fig. 2c). However, the increased Co/P atomic ratio by EDX and inductively coupled plasma-optical emission spectrometry (ICP-OES) means the content of P species has been decreased remarkably because of the possible P leaching (Fig. S3d†).

**Fig. 2:**
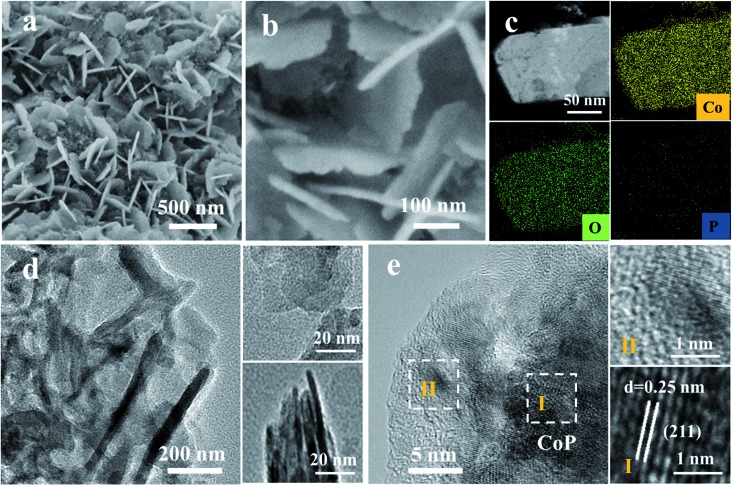
(a and b) SEM images, (c) HAADF-TEM image and EDX elemental mapping images, and (d and e) TEM images of CoP-A.

The HER electrocatalytic activities are evaluated in 1.0 M KOH electrolyte with a graphite rod as the counter electrode. For comparison, its Co_3_O_4_ counterpart and Pt/C are also benchmarked under identical conditions. During the electrochemical activation, the potential demonstrates an apparent decreasing trend (Fig. 3a), which suggests that this activation is effective to lower the potential and enhance the activity. The initial CoP nanosheets exhibit an activity with an onset potential of 90 mV and an overpotential of 180 mV to reach 10 mA cm^–2^ current density. After activation, CoP-A shows a significantly enhanced electrocatalytic performance, specifically a much smaller onset potential of 30 mV and a lower overpotential of 100 mV to afford 10 mA cm^–2^ current density (Fig. 3b). Moreover, the exchange current density (*j*_0_) of CoP-A is 0.67 mA cm^–2^, almost double that of CoP (0.36 mA cm^–2^) and much higher than that of its Co_3_O_4_ counterpart (0.05 mA cm^–2^) (Table S1†), indicating an improved intrinsic activity of CoP-A.^30^,^31^ The resulting HER activity of CoP-A is also better than that of most of the recently reported cobalt-based or phosphor-based HER electrocatalysts in alkaline solution (Table S2†), and even comparable to that of commercial Pt/C over a larger potential range (*ca.* –0.32 V). Furthermore, a lower Tafel slope of 76 mV dec^–1^ for CoP-A than that of CoP (95 mV dec^–1^) reveals an improved kinetics for hydrogen evolution (Fig. 3c). The excellent electrochemical stability is another good characteristic of the CoP-A catalyst. No evident driving potential fluctuation for CoP-A catalysts is observed during 25 h of operation at an overpotential of 120 mV (Fig. 3d inset), and the activity remains the same even after 5000 potential cycles (Fig. 3d). Electrochemical impedance spectroscopy (EIS) results in Fig. 3e show that the CoP-A catalyst has the smallest charge transfer resistance (*R*_ct_), suggesting the improved rate of ion exchange and electron transfer at the reaction surface/interface of electrolyte and catalysts.^32^ Double layer capacitance (*C*_dl_) is usually adopted to calculate indirectly the electrochemical surface area (ECSA) of catalysts. The double layer capacitance (*C*_dl_) of CoP-A is 5.1 mF cm^–2^ (Fig. 3f), which is two times that of CoP (2.5 mF cm^–2^) and much higher than that of the Co_3_O_4_ precursor (0.95 mF cm^–2^), corresponding to the highest ECSA of CoP-A among the three compared electrocatalysts (Table S1†). Moreover, the turnover frequency (TOF) of CoP-A is calculated to be 0.234 s^–1^ at an overpotential of 100 mV, which is much higher than that of CoP (0.051 s^–1^) and Co_3_O_4_ (0.016 s^–1^), suggesting a higher intrinsic activity of CoP-A than that of CoP and Co_3_O_4_ (Table S1†).^33^

**Fig. 3:**
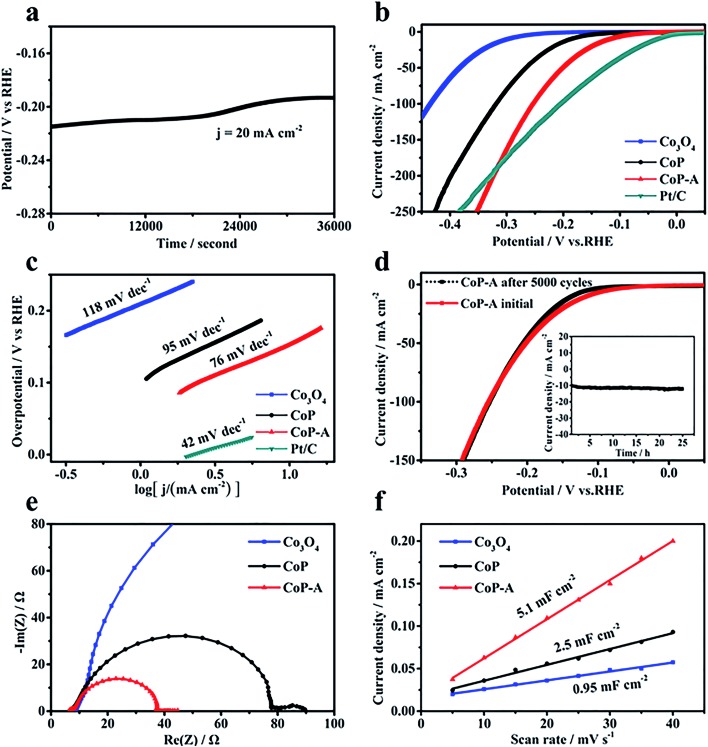
(a) Electrochemical activation at 20 mA cm^–2^; (b) LSV curves and (c) Tafel plots of Co_3_O_4_, CoP, CoP-A and Pt/C (20%) in 1.0 M KOH solution (90% *iR* corrected); (d) LSV curves of CoP-A before and after 5000 cycles (inset: long-term stability test of CoP-A at the 120 mV overpotential); (e) EIS curves of Co_3_O_4_, CoP and CoP-A; and (f) double layer capacitance of Co_3_O_4_, CoP and CoP-A.

The evolution of the surface chemical composition and valence of CoP samples are investigated by the X-ray photoelectron spectroscopy (XPS) technique (Fig. S6a†). A typical pair of Co 2p_3/2_ and Co 2p_1/2_ is responsible for the Co 2p spectrum of CoP (Fig. 4a).^34^,^35^ The Co 2p_3/2_ region exhibits two main peaks at 778.6 and 781.6 eV with one satellite peak at 786.2 eV, while the Co 2p_1/2_ region also has two main peaks at 793.7 and 797.7 eV with a satellite at 802.7 eV. The main peaks at 778.6 and 793.7 eV for Co 2p are close to those of Co(0), corresponding to Co^*δ*+^ (0 < *δ* < 1) in CoP.^35^ In addition, the P 2p XPS spectrum exhibits two peaks at 129.7 and 130.6 eV (Fig. 4b), corresponding to P 2p_3/2_ and P 2p_1/2_, respectively.^35^ Another two evident peaks at 131.9 and 134.1 eV are attributed to the oxidized P species due to the surface oxidation when exposed to air.^36^,^37^ However, a significant difference is observed when comparing the XPS spectrum of CoP-A with that of the initial CoP sample. The binding energy of Co 2p in CoP-A displays a positive shift (Fig. 4a). The peak intensity of Co^*δ*+^ for CoP-A at 778.6 eV in Co 2p spectra decreases substantially and the peak at 793.7 eV disappears after the activation, while the peak at 781.1 eV is assigned to oxidized Co.^31^,^38^ Meanwhile, the presence of two emerging peaks at 783.0 eV and 786.0 eV suggests the formation of new species on the surface of CoP-A. It can be concluded that Co species is mainly transformed into low valence Co oxides or hydroxides rather than metallic Co or high valence Co compounds. For the spectrum of P 2p, the peak at 131.9 eV for CoP-A disappeared, while the presence of a peak at 134.1 eV is attributed to orthophosphate (Fig. 4b). The Fourier-transform infrared spectroscopy (FT-IR) result in Fig. 4c verifies the emergence of –OH species in the activated CoP-A sample.^36^,^39^ Apart from the peaks at 225, 281, 296 and 318 cm^–1^, which are attributed to cobalt phosphide, the Raman spectrum of CoP-A also shows the presence of peaks at 512 cm^–1^ and 691 cm^–1^, which are assigned to the Co–O stretching mode (Fig. S7†). Moreover, the new species formed should be dispersed homogeneously on the surface of CoP nanosheets because the elements are dispersed evenly according to the elemental mapping images of CoP-A (Fig. 2c) and no obvious aggregation is observed. The above results indicate that the structure and composition of CoP catalysts experience irreversible and significant changes during the activation process. Such surface evolution in alkaline solution would be due to P leaching from the CoP nanosheets during the electrochemical activation, which is consistent with the EDX and ICP-OES results (Co 48.2 at% and P 8.1 at%, Fig. S3d†).^37^,^38^

**Fig. 4:**
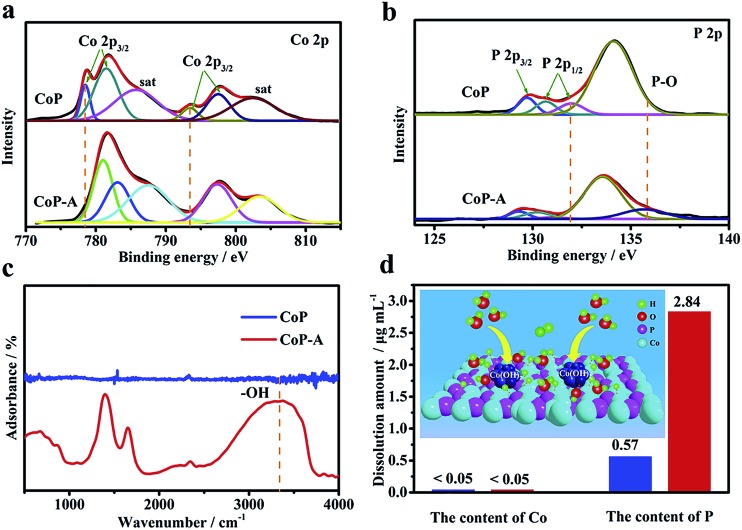
High-resolution Co 2p (a) and P 2p (b) XPS spectra, and (c) FT-IR spectra of CoP and CoP-A. (d) The dissolution amount of Co and P in the KOH electrolyte (inset: schematic diagram of the HER process).

In order to further confirm the result, the electrolytes after electrochemical tests and controlled immersion experiments are collected and analyzed by ICP-OES (Fig. 4d). When the catalysts are only soaked in the electrolyte solution for 10 hours, the solution contents of Co and P species are relatively low with less than 0.05 μg mL^–1^ and 0.57 μg mL^–1^ for Co and P, respectively. However, Co still remains at a low concentration (<0.05 μg mL^–1^), while the concentration is more than 2.84 μg mL^–1^ for P after activation in the electrolytes, quintupling the initial CoP sample. This significant change of the surface P content again confirms that the bare CoP nanosheets suffer from substantial dissolution of P and consequently form cobalt (oxy)hydroxide rather than the simple dissolution/corrosion of CoP in a stoichiometric ratio. The surface evolution of the CoP catalyst from stoichiometric Co–P to the Co-rich surface can be explained as a result of the replacement of polyphosphates by Co-based hydroxides because of the corrosion/dissolution of polyphosphate species.^36^,^40^ Therefore, it can be concluded that the surface P atoms are leached resulting in the exposure of adjacent Co atoms, and the nonstoichiometric Co is active and could easily combine with OH^–^ in the electrolyte, consequently resulting in *in situ* formation of Co-based species Co(OH)_*x*_ and the final stable Co(OH)_*x*_@CoP hybrid.

The formed stable Co(OH)_*x*_@CoP hybrid demonstrates an enhanced activity with an improved kinetics for the HER. The decreased Tafel slope was 76 mV dec^–1^ (Fig. 3c), indicating that the reaction still mainly consists of Volmer and Heyrovsky steps.^18^,^19^ CoP can act as a promising HER electrocatalyst due to its strong ability to adsorb hydrogen to facilitate the Volmer step and consequently accelerate the hydrogen evolution.^12^ In the alkaline electrolyte, however, the dissociation of water rather than the adsorption of hydrogen is the rate determining step.^41^,^42^ Co(OH)_*x*_ species have been proved to have a strong ability toward water dissolution, which can capture the water molecular with a fast rate and offer abundant hydrogen atoms *via* the dissociation.^27^,^28^ As illustrated in the Fig. 4d inset, the *in situ* formed Co(OH)_*x*_ accelerates the water dissociation, which leads to abundant hydrogen atoms, and then the H atoms transfer to the nearby exposed active sites of CoP to form Co–H bonds, followed by recombination with water to produce hydrogen molecules. This is consistent with the experimental reaction pathway derived from the Tafel slope. For comparison, Co(OH)_2_ nanosheets are also prepared and show poor HER activity (Fig. S4†), further confirming that the high performance of Co(OH)_*x*_@CoP is derived from the synergistic effect arising from both Co(OH)_*x*_ and CoP. Moreover, the surface Co(OH)_*x*_ formation would consume the OH^–^ species at the local surface, which can further accelerate the detachment of adsorbed OH^–^ on the surface. Therefore, the catalytic performance will increase as the activation proceeds until it reaches a steady state, which is consistent with our electrochemical measurements (Fig. 3).

In summary, an *in situ* electrochemical strategy is developed to effectively reconstruct the surface of CoP nanosheets in alkaline media. The final product, the Co(OH)_*x*_@CoP hybrid, shows an enhanced HER activity with an overpotential of 100 mV at 10 mA cm^–2^ and a Tafel slope of 76 mV dec^–1^, which are better than those of most CoP-based electrocatalysts in alkaline electrolyte. The electrocatalytic performance enhancement of the Co(OH)_*x*_@CoP hybrid nanosheets is mainly derived from the evolution and reconstruction of the surface structure and composition. The formation of low-valence Co compounds of Co(OH)_*x*_ on the surface of CoP can effectively increase its ability for water dissociation, further boosting the HER kinetics. This work not only offers an effective strategy for the rational design of functional electrocatalysts, but also provides useful understanding of the surface evolution of catalysts and broadens our horizon to explore more efficient catalysts for practical application.

## Conflicts of interest

There are no conflicts to declare.

## Supplementary Material

Supplementary information
