# Supplementary material for: Surface reconstruction of cobalt phosphide nanosheets by electrochemical activation for enhanced hydrogen evolution in alkaline solution
Source: Chem Sci. 2018 Dec 6;10(7):2019–24. doi: 10.1039/c8sc04589e (PMC6375356; doi:10.1039/c8sc04589e)
Supplement: Supplementary file 1 [file SC-010-C8SC04589E-s001.pdf]

Electronic Supplementary Material (ESI) for Chemical Science.
This journal is © The Royal Society of Chemistry 2018

Electronic Supplementary Information

Surface Reconstruction of CoP Nanosheets by Electrochemical Activation for Enhanced Hydrogen Evolution in Alkaline Solution

Liang Su, [1] Xiangzhi Cui, [2,] Ting He, [1] Liming Zeng, [2,3] Han Tian, [2,3] Yiling Song, [2,3] Kai Qi, [1] and Bao Yu Xia [1,*]*

[1]Key Laboratory of Material Chemistry for Energy Conversion and Storage (Ministry of Education), Hubei Key Laboratory of Material Chemical and Service Failure, Wuhan National Laboratory for Optoelectronics, School of Chemistry and Chemical Engineering, Huazhong University of Science and Technology (HUST), 1037 Luoyu Road, Wuhan 430074, PR China

[2]State Key Laboratory of High Performance Ceramics and Superfine Microstructures, Shanghai Institute of Ceramics, Chinese Academy of Sciences, Shanghai 200050, PR China

[3]University of the Chinese Academy of Sciences, Beijing 100049, PR China

Corresponding author: byxia@hust.edu.cn (B. Y. Xia); cuixz@mail.sic.ac.cn (X. Z. Cui)

Experimental section

Materials: Cobaltous acetate tetrahydrate, glycerol, ethylene glycol, ethanol, potassium hydroxide, sodium hypophosphite and all chemicals involved were all purchased from Aladdin. Nafion solution (5 wt%) was obtained from Sigma-Aldrich. All chemical reagents were used without any further purification.

Preparation of CoP: For the synthesis of CoP, 800 mg of cobaltous acetate tetrahydrate were dispersed in 40 mL of glycerol solution with the assistance of magnetic stirring. After stirring 2 hours, the well mixture was transferred into an 80 mL Teflon-lined stainless steel autoclave. The vessel was sealed and heated in an oven at 180 °C for 4 hours, and then cooled to room temperature naturally. The products were purified via centrifugation with ethanol several times and evaporated at 60 °C for 24 hours to get a dried powder. Then the collected powders were calcined in air at 400 °C for 2 hours with a heating rate of 5 °C per minute. Next, the products and NaH_2PO_2 were placed at two separate quartz boats with NaH_2PO_2 boat at the upstream side, and heated to 350 °C with a ramp rate of 5 °C per minute in H_2/Ar (5% H_2) atmosphere and maintained for 2 hours. The CoP was finally obtained after cooled down to room temperature.

Preparation of $Co(OH)_2$ nanosheets: The $Co(OH)_2$ nanosheets were typically synthesized by a hydrothermal process, 1000 mg cobaltous acetate tetrahydrate were dissolved into 60 mL solution consisting of 55 mL ethylene glycol and 5 mL deionized water under continuous magnetic stirring. Then, the well mixed solution was transferred into an 80 mL Teflon-lined stainless steel autoclave and sealed. The vessel was heated in an oven at 200 °C for 8 hours, and then cooling down to room temperature. Finally, the light green products were collected by centrifugation, washed with ethanol three times and evaporated at 60 °C for 24 hours.

Characterizations

X-ray diffraction measurements were carried on a Rigaku D/Max 2200PC XRD system (Cu Kα radiation) at 40 mA and 40 kV. Low and high resolution transmission electron microscopic images and energy-dispersive X-ray spectrometry (EDX) elemental mapping were performed on a JEOL 200CX electron microscope under 200 kV. Scanning Electron Microscope (SEM) images were obtained on FEI Magellan 400 instrument. X-ray photoelectron spectroscopy (XPS) measurement was conducted out on Thermo

Scientific ESCALAB 250 spectrometer with AI Kα radiation as the excitation source. Binding energies for the high resolution spectra were calibrated by setting C 1s to 284.6 eV. Fourier transformed infrared (FT-IR) spectra were recorded with a Nicolet IS10 FT-IR spectrometer. The concentration of elements was measured on inductively couple plasma optical emission spectroscopy (ICP-OES, Agilent Technologies). Raman spectrum were collected using a Renishaw in-Via Raman microprobe equipped with a Leica DM 2500 M microscope system. Raman spectrum were excited by a 523 nm diode-pumped laser at a resolution of 4 cm^{-1} in the range between 100-4000 cm^{-1}.

Electrochemical measurements: For the preparation of working electrodes, 8.0 mg of CoP powders and 30 μL of Nafion (5 wt%) were dispersed in 1 mL of ethanol and water (1:1 in volume). The mixture was ultrasonicated for at least 30 minutes to generate a homogeneous ink, then 8 μL of ink was dropped onto the glassy carbon electrode with a diameter of 5 mm. The amount of CoP catalyst deposited on the glassy carbon electrode was controlled as 0.4 mg cm^{-2}. All electrochemical measurement was performed at room temperature by using a CHI 760E electrochemical workstation (CH Instrument, Shanghai, China) with a standard three-electrode cell. An Ag/AgCl in 3M KCl solution acted as the reference electrode, and a graphite rod (6 mm in diameter) was used as counter electrode. An aqueous solution of 1.0 M KOH (pH = 14) was used as the electrolyte. All potentials were referenced to a reversible hydrogen electrode (RHE) with 90% *i*R correction: $E_{vs\ RHE} = E_{vs.Ag/AgCl} + 0.0591*pH + E_{Ag/AgCl}^{\theta}$ (0.209) - *i*R, where the R was referred to the average ohmic resistance obtained from EIS measurement.[1] The *in-situ* surface reconstruction was carried out by chronpotentiometry under a constant current density of 20 mA cm^{-2} for 10 hours in 1.0 M KOH. Electrochemical tests of CoP-A were conducted after the V-t curves becoming stable. The scan rate of cyclic voltammetry (CV) is 0.05 V s^{-1}, while that is 0.005 V s^{-1} for linear sweep voltammetry (LSV). The Tafel plots were obtained by fitting the polarization curves as overpotential η versus log current density (log[j]). Electrochemical impedance spectroscopy (EIS) was performed from 100 KHZ to 0.01 HZ at -150 mV (vs RHE) with an amplitude of 5 mV. Cyclic voltammetry (CV) was conducted to measure the electrochemical double layer capacitance (EDLC) at non-Faradaic potential ranging from 0.15 to 0.35 V (vs. RHE) with various scan rates (5, 10, 15, 20, 25, 30, 35, 40 mV s^{-1}), which can estimate the effective electrochemical surface area. The long-stability of catalysts was also tested by chronoamperometry, which

was carried out at constant overpotential of 120 mV for 25 hours. A flow of N_2 was maintained during the experiment.

The evaluation of main electrochemical parameters (b, j_o, C_{dl}, ESCA, TOF).[2]

Tafel slope (b) and exchange current density (j_0): Tafel plots can be obtained by fitting the polarization curves as overpotential versus log current density: $\eta = a + b\log[j]$, η is overpotential, j is current density and b is Tafel slope, which obtained by fitting in the linear region of Tafel curve and used to indicate the characteristics of the hydrogen evolution reaction kinetics. Exchange current density (j_0) is the intersection of the Tafel curve in the extension in linear region, which also reveals the kinetics of hydrogen evolution reaction and the intrinsic activity of electrocatalysts. Electrochemical double layer capacitance (C_{dl}) and electrochemical active surface area (ECSA). The double layer capacitance evaluated by cyclic voltammetry in non-Faradaic region at various scan rates, the capacitive current density ($|j_a - j_c|/2$) is proportional to the scan rate (v). Moreover, the electrochemical active surface area is calculated from the electrochemical double layer capacitance: $S_{ESCA} = C_{dl}/C_s$, where C_s is the specific capacitance of the material or the capacitance of an atomically smooth planar surface of the material per unit area under identical electrolyte conditions, here, we adopt general specific capacitances of $C_s = 0.04$ mF cm^{-2} in 1.0 M KOH. The turnover frequency (TOF) was calculated according to the equation: TOF=$\frac{jS}{2nF}$, Where j is the current density at an overpotential of 100 mV, S is the geometric surface area of GC electrode (0.196 cm^2), 2 stands for a two electron reaction process in HER, F is Faraday constant (96,500 mol/C), n is the number of moles of metal atoms deposited onto the GC electrodes that calculated from mass loading.[3]

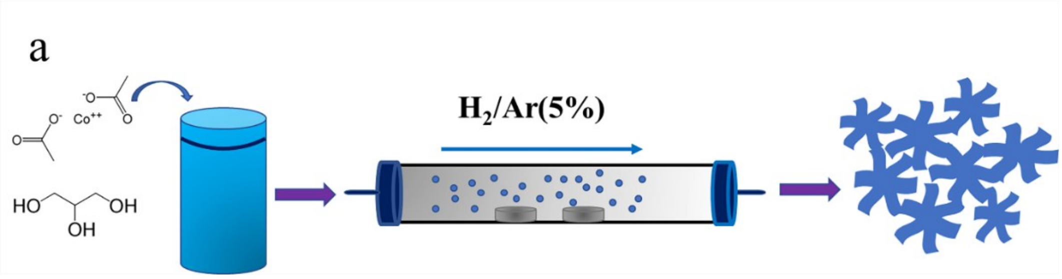

Figure S1. Schematic synthesis of CoP nanosheets.

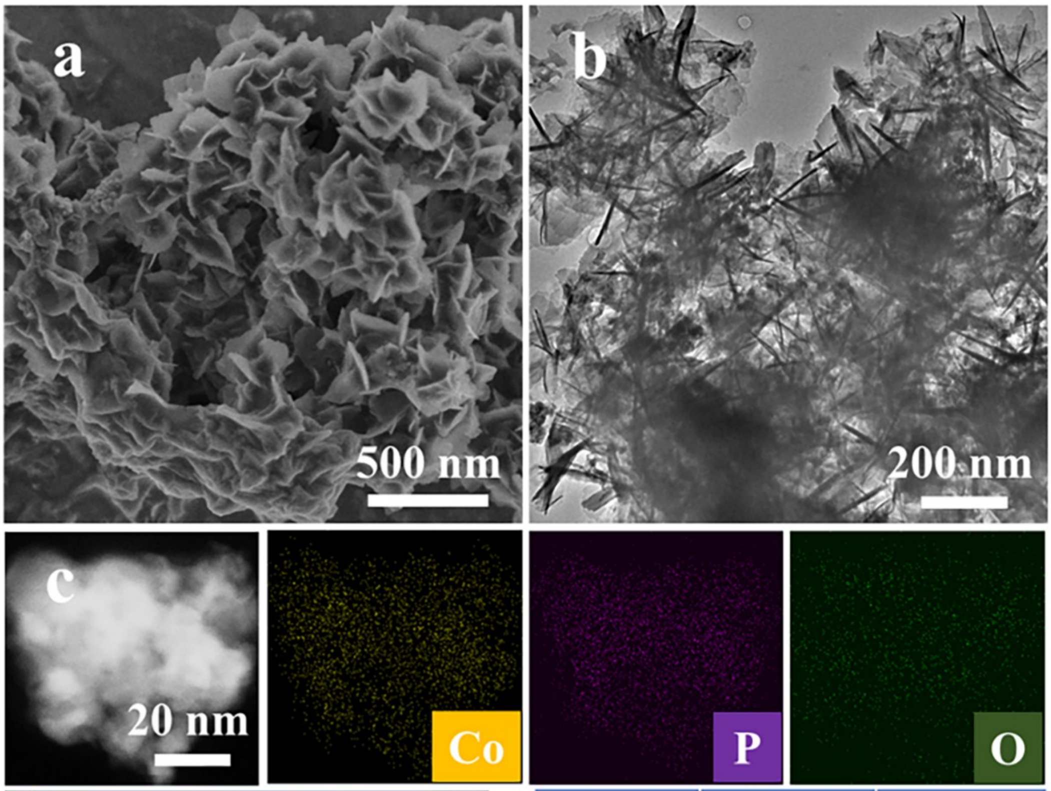

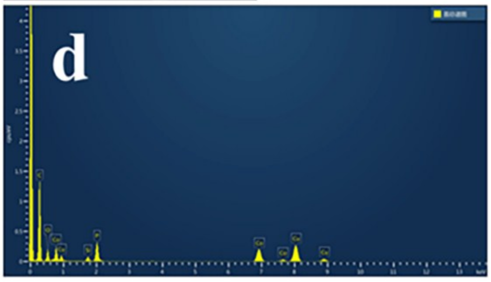

e

Element	Wt%	Atomic %
O	34.18	59.70
P	21.27	19.18
Co	44.55	21.12

Figure S2. SEM (a) and TEM (b) image of CoP, (c) HAADF-TEM image and elemental mapping of CoP, (d) EDX pattern and (e) elemental contents of CoP.

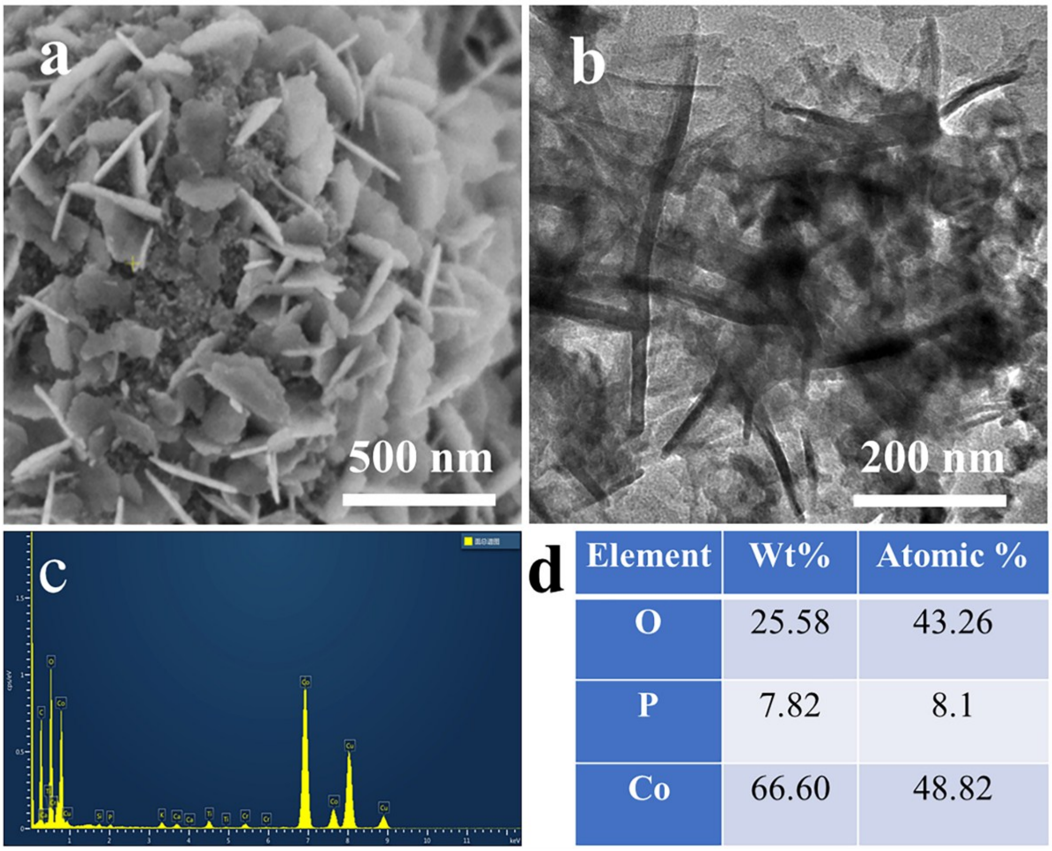

Element	Wt%	Atomic %
O	25.58	43.26
P	7.82	8.1
Co	66.60	48.82

Figure S3. SEM (a) and TEM (b) image of CoP-A; (c) EDX pattern and (d) elemental contents of CoP-A.

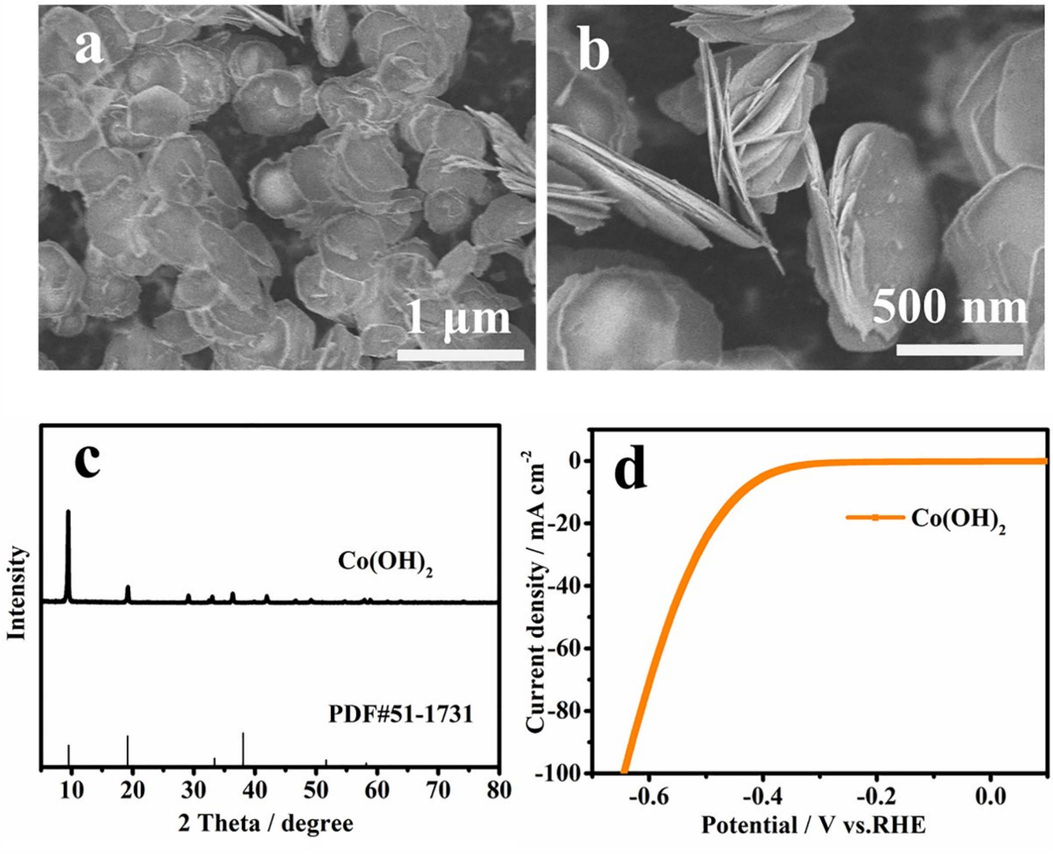

Figure S4. (a, b) SEM images, (c) XRD patterns and (d) LSV curve of reference $Co(OH)_2$.

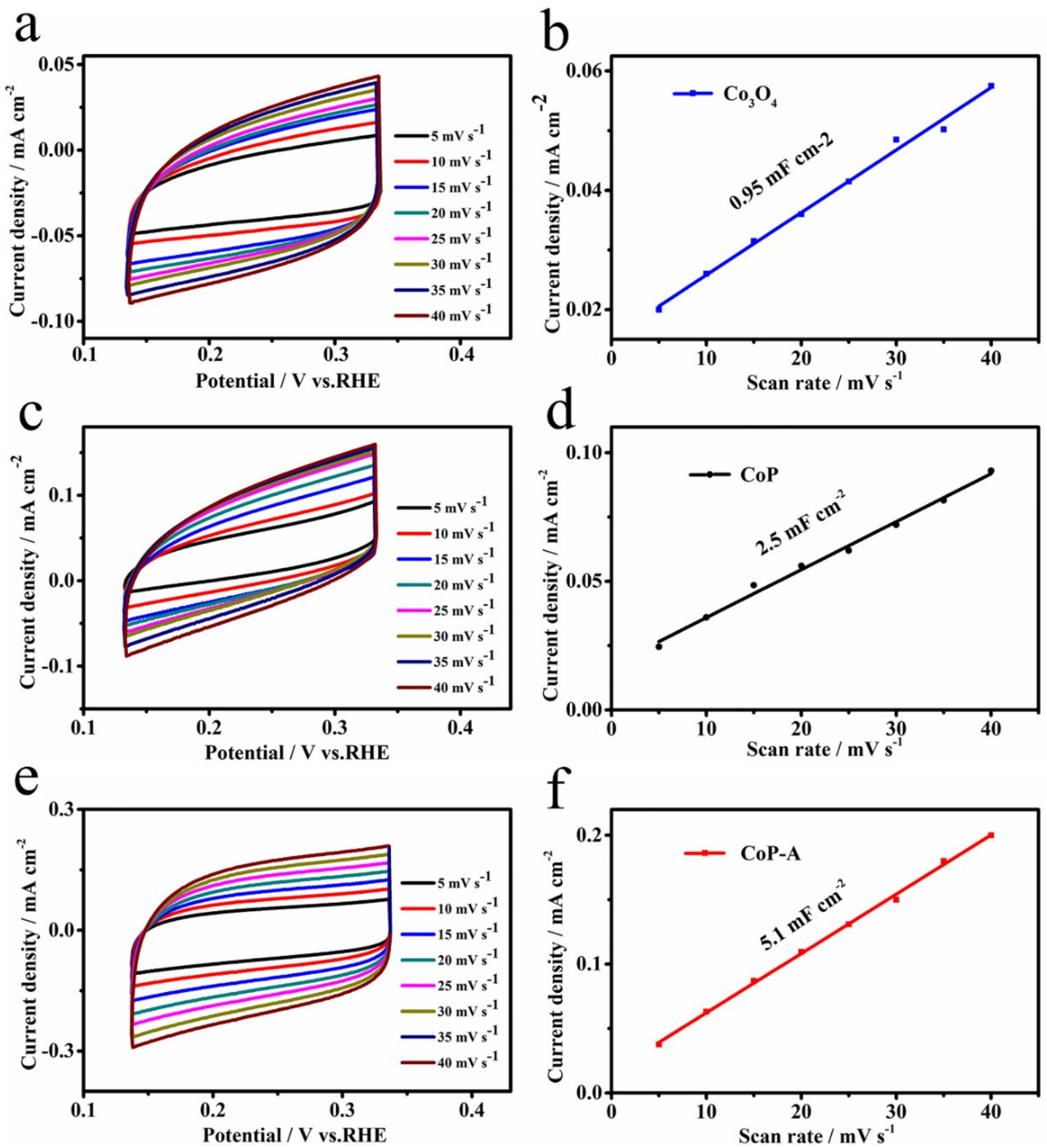

Figure S5. Non-faradaic CV scans of Co_3O_4 (a), CoP (b) and CoP-A (c) at different scan rate and the corresponding double layer capacitance.

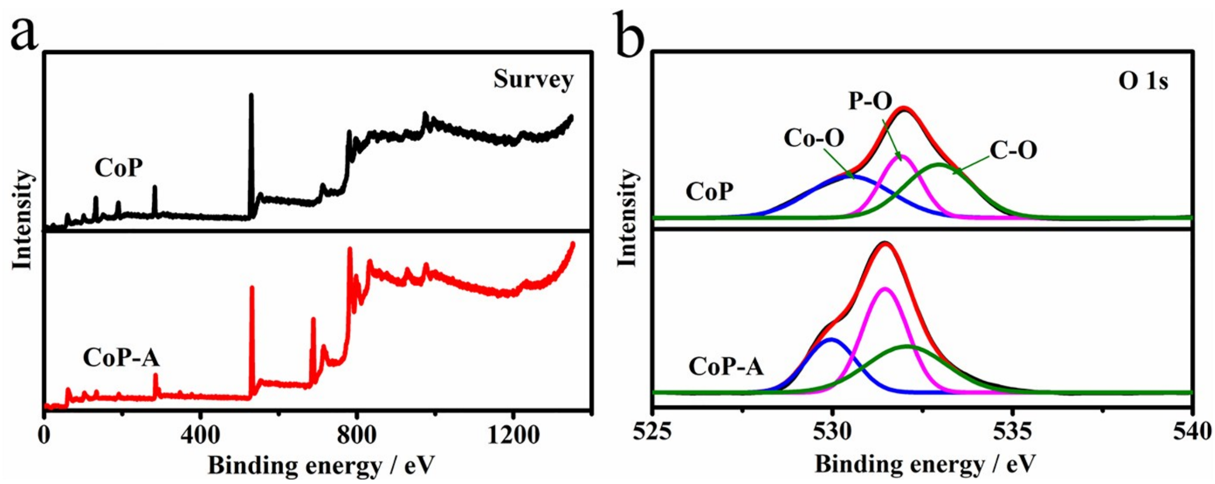

Figure S6. (a) full XPS survey and (b) high resolution O 1s XPS spectra of CoP and CoP-A.

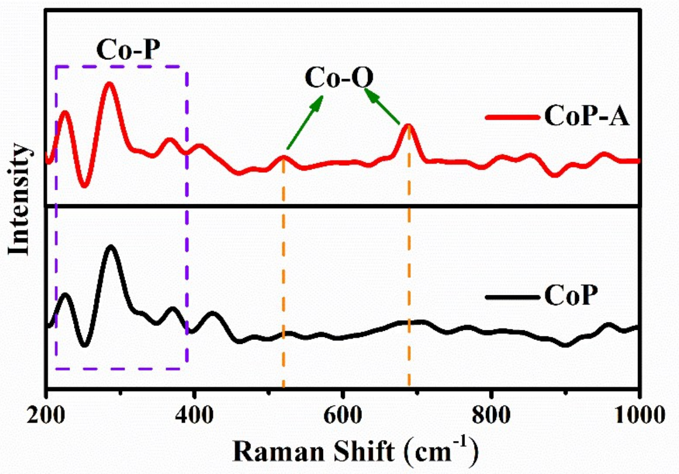

Figure S7. Raman spectra of CoP and CoP-A.

Table S1. Electrochemical activity comparison of three samples.

	Ms[1]	η_{on}[2]	η_{10}[3]	b[4]	Cd[5]	S_0[6]	J_0 [7]	TOF[8]
**CoP-A**	0.4	30	100	76	5.1	127.5	0.67	0.234
**CoP**	0.4	90	180	95	2.5	62.5	0.36	0.051
**Co_3O_4**	0.4	210	300	118	0.95	23.8	0.05	0.016

Ms[1]: Mass loading of catalyst (mg cm^{-2});

η_{on}[2]: Onset overpotential (mV);

η_{10}[3]: Overpotential of current density at 10 mA cm^{-2} (mV);

b[4]: Tafel slope derived from LSV (mV dec^{-1});

Cd[5]: Double layer capacity at non-faradic region (mF cm^{-2});

S_0[6]: Electrochemical surface area (cm^2);

J_0 [7]: Exchange current density (mA cm^{-2});

TOF[8]: Turnover frequency (s^{-1}).

Table S2. HER performances comparison of recent electrocatalysts in alkaline medium (1.0 M KOH).

Catalysts	Ms[1]	η_{on}[2]	η_{10}[3]	b[4]	TOF[5]	Reference
CoP/Ti	1.03	40	128	78	*NA*	*Adv. Mater. 2017, 29, 1602441*
CoP nanowire/CC	0.92	30	106	51	0.725 (75 mV)	*J. Am. Chem. Soc. 2014, 136, 7587*
$Ni_{0.23}Co_{0.77}P$ films	1.0	40	128	65	*NA*	*Adv. Funct. Mater. 2016, 26, 7644*
CoS_2 NTA/CC	1.2	110	193	81	*NA*	*Nanoscale Horiz. 2017, 2, 342*
CoP/CFP	1.0	70	128	50	*NA*	*ACS Appl. Mater. Interfaces 2018, 10, 14777*
CoP/NCNHP	0.25	95	115	66	0.11 (100 mV)	*J. Am. Chem. Soc. 2018, 140, 2610*
CoP@NPMG	0.2	50	151	75	*NA*	*Nanoscal 2018, 10, 2603*
CoP/C	0.5	100	151	128	0.125 (100 mV)	*Electrochim. Acta. 2017, 11, 040*
CoP nanowire	0.8	90	137	69	0.15 (150 mV)	*Int. J. Hydrogen Energy* ***2017, 42, 29080***
CoP-C	0.84	70	163	63	*NA*	*J. Energ. Chem. 2017, 26, 1147*
$CoSe_2$	1.02	75	103	69	14.95 (200 mV)	*Nat. Commun. 2018, 9, 2533*
FeCoP	1.0	50	120	76	*NA*	*Nano Energy 2017, 41, 583*
**CoP-A**	**0.4**	**30**	**100**	**76**	**0.234 (100 mV)**	**This work**

Ms[1]: Mass loading of catalyst (mg cm^{-2});

η_{on}[2]: Onset overpotential (mV);

η_{10}[3]: Overpotential of current density at 10 mA cm^{-2} (mV);

b[4]: Tafel slope derived from LSV (mV dec^{-1});

TOF[5]: Turnover frequency (s^{-1}).

Note: *NA* means no available.

References

1. Y. Zhou, W. Huang, X. Zhang, M. Wang, L. Zhang and J. Shi, *Chem. Eur. J.,* 2017, **23**, 17029-17036.

2. S. Anantharaj, S. R. Ede, K. Karthick, S. Sam Sankar, K. Sangeetha, P. E. Karthik and S. Kundu, *Energy Environ. Sic.,* 2018, **11**, 744-771.

3. J. Tian, Q. Liu, A. M. Asiri and X. Sun, *J. Am. Chem. Soc.,* 2014, **136**, 7587-7590.

4. A. Goryachev, L. Gao, Y. Zhang, R. Y. Rohling, R. H. J. Vervuurt, A. A. Bol, J. P. Hofmann and E. J. M. Hensen, *ChemElectroChem,* 2018, **5**, 1230-1239.

5. J. Yang, H. Liu, W. N. Martens and R. L. Frost, *J. Phys. Chem. C,* 2010, **114**, 111-119.
